# Exploring the Impact of Solid-State Fermentation on Macronutrient Profile and Digestibility in Chia (*Salvia hispanica*) and Sesame (*Sesamum Indicum*) Seeds

**DOI:** 10.3390/foods11030410

**Published:** 2022-01-30

**Authors:** Joaquim Calvo-Lerma, Andrea Asensio-Grau, Jorge García-Hernández, Ana Heredia, Ana Andrés

**Affiliations:** 1 Instituto de Ingeniería de Alimentos para el Desarrollo, Universitat Politècnica de València, 46022 València, Spain; joaquim_calvo@iata.csic.es (J.C.-L.); anhegu@tal.upv.es (A.H.); aandres@tal.upv.es (A.A.); 2 Instituto de Agroquímica y Tecnología de Alimentos, Spanish Scientific Research Council, 28006 Madrid, Spain; 3 Centro Avanzado de Microbiología de Alimentos, Universitat Politècnica de València, 46022 València, Spain; jorgarhe@btc.upv.es

**Keywords:** chia seeds, sesame seeds, *Pleurotus ostreatus*, solid-state fermentation, lipolysis, proteolysis, viscosity, fatty acids

## Abstract

Fermentation of plant-based substrates with edible fungi enhances the nutrient profile and digestibility, but it has been scarcely applied to edible seeds, which are rich in healthy lipids. In this study, chia and sesame seeds were solid-state fermented with *Pleurotus ostreatus*, followed by drying and milling. Fermentation led to increased content of lipid and protein in both seeds’ products, and a change in fatty acid profile in favor of increased polyunsaturated fatty acids. Then, the samples were subjected to in vitro digestion. Lipolysis, determined by nuclear magnetic resonance, was higher in sesame than in chia products, and the fermented counterparts had increased values compared to the controls. In terms of physical properties, fermentation showed reduced particle size and increased matrix degradation and decreased viscosity of the digestion medium, which were related to increased lipolysis. In conclusion, applying solid-state fermentation on chia and sesame seeds could be a recommendable approach.

## 1. Introduction

In developed industrialized countries, unhealthy dietary habits are mainstream. Market availability and consumers’ preference for unhealthy food products have led to excessive intakes of saturated fat and deficient sources of fiber and unsaturated fatty acids [1]. This scenario is promoted by consumers’ lack of time for cooking, unhealthy foods being cheaper than recommendable foods, and the scarce availability of healthier and affordable options. This situation will keep increasing obesity and rates of non-communicable diseases [2]. In contrast, health authorities recommend plant-based foods to balance the diet towards higher intakes of vegetal-protein, fiber and bioactive compounds [3].

Among the many innovations in the food industry in recent decades, some have contributed to the situation described above, while others can be considered as allied strategies to revert it. Fermentation is known for being applied in the production of yoghurts, cheese and alcoholic beverages, but also, several plant-origin by-products have been fermented to increase sensory or technological qualities [4]. Taking this background, the technique is being applied to improve the nutrient profile of cereals and legumes, which result in reduced anti-nutritional factors (ANFs) along with increased protein content and antioxidant activity [5].

Concerning seeds, fermentation has been applied to a lesser extent than in legumes or cereals [4]. However, this vegetal group could largely benefit from this biotechnological process. While seeds present with an excellent nutrient profile, which includes high fiber, bioactive compounds, protein and healthy fat, the physical structure of the shell is hardly disrupted during the digestion process, thus impairing the release of these nutrients and hindering their further bioabsorption [6,7]. So, the shell structure should be disrupted with the aim of making seeds’ nutrients more bioaccessible. Previous research has focused on assessing pre-treatments prior to digestion to improve digestibility of protein and lipids in chia seeds, with only milling having proved to have an enhancing effect, and hydration or mastication having been shown to have little to no effect [6]. In this sense, fermentation could contribute to the degradation of the shells of the seeds, favoring subsequent nutrient release and digestibility, as the components of the shell are used by the fermenting agents as substrates [8].

In previous research, the use of *Pleurotus ostreatus*, an edible fungus, has been applied in vegetal-origin substrates, such as lentils, beans or peas, resulting in improved nutrient profile and digestibility, and desirable organoleptic characteristics after drying and milling for the purpose of obtaining stable fermented flours [4]. Some authors have pointed to fermented flours as potential ingredients in the formulation of novel foods, with a balanced nutrient profile that contributes to healthier diets and helps to prevent diet-related pathologies [9,10]. However, the examples mentioned above do not include substrates that are rich in lipids with unsaturated fat profiles, as in chia or sesame seeds.

Thus, the aim of the present study was to analyze the potential of fungal solid-state fermentation in edible and lipid-rich substrates (chia and sesame seeds) to obtain fermented products with enhanced nutrient profiles, antioxidant properties and digestibility.

## 2. Materials and Methods

### 2.1. Materials

Chia (*Salvia hispanica*) and sesame (*Sesamum indicum*) seeds were purchased at a local supermarket in Valencia (Spain). The strain of Pleurotus ostreatus was acquired at the Spanish Culture Type Collection (CECT) (20311/Batch 18-10-2016). The culture broth was formulated with a mix of yeast, malt, glucose and agar extracts (Scharlab, Barcelona, Spain). To simulate digestion, the digestive fluids were prepared with the following reagents: pepsin from porcine gastric mucosa (2500 U g^−1^ protein), pancreatin from porcine pancreas extract (8 × USP), bovine bile extract, potassium chloride (KCl), potassium dihydrogen phosphate (KH_2_PO_4_), sodium hydrogen carbonate (NaHCO_3_), sodium chloride (NaCl), magnesium chloride hexahydrate (MgCl_2_·6H_2_O), ammonium carbonate ((NH_4_)2CO_3_) and calcium chloride (CaCl_2_), all from Sigma-Aldrich (St Louis, MO, USA), and sodium hydroxide (NaOH) and hydrogen chloride (HCl) from AppliChem Panreac (USA). To carry out the analytical determinations, the following were required: methanol (CH_3_OH), ethanol (CH_3_CH_2_OH), bovine seroalbumin, gallic acid, Trolox (C_14_H_18_O_4_), DPPH (2,2-diphenyl-1-picrylhydrazyl), iron (III) chloride hexahydrate (FeCl_3_·6H_2_O), TPTZ (2,4,6-Tris(2-pyridyl)-s-triazine), acetic acid (C_2_H_4_O_2_), ABTS (C_18_H_24_N_6_S_4_), potassium persulfate (K_2_S_2_O_8_), trichloroacetic acid (TCA), dinitrosalicylic acid (DNS), glucose (C_6_H_12_O_6_), sodium potassium tartrate (C_4_H_4_KNaO_6_), invertase, dichloromethane (CH_2_Cl_2_) and deuterated chloroform (CHCl_3_) (Sigma-Aldrich; Barcelona, Spain), ethylenediaminetetraacetic acid (EDTA; C_10_H_16_N_2_O_8_) and crystalline urea (NH_2_CONH_2_) for analysis (ACS), (AppliChem Panreac, Chicago, IL, USA) and amylogucosidase (Megazyme, Co. Wicklow, Ireland).

### 2.2. Solid-State Fermentation with Pleurotus Ostreatus

Solid-state fermentation was conducted according to the methodology followed by Asensio-Grau et al. (2020) in lentils [5]. *Pleurotus ostreatus* colonies were taken from the original batch and cultured in petri dishes with a broth comprised of 4% (*w*/*v*) yeast extract, 0.1% (*w*/*v*) malt extract, 0.4% (*w*/*v*) glucose and 1.5% (*w*/*v*) agar and incubated at 28 °C for 14 days (Selecta J.P.200207, Germany) for mycelium growth. Then, it was recovered with an inoculating loop and introduced in a flask containing the liquid broth (4% (*w*/*v*) yeast extract, 0.1% (*w*/*v*) malt extract and 0.4% (*w*/*v*) glucose), and incubation continued at 28 °C until 264 mg of dry biomass was obtained in 100 mL of broth. The dry biomass was considered as the weight of the mycelium after filtering and drying until reaching a constant weight. At this point, the fungus was ready for inoculating the substrates.

For fermentation, chia or sesame seeds (70 g) were introduced in 1 L glass jars, mixed with water in a proportion 1:0.75 and autoclaved at 121 °C for 20 min (in triplicate). Then, 8 mL of the liquid broth containing the mycelium, previously vortexed, were inoculated in the jars. Fermentation was allowed for 14 days in incubation at 28 °C. The control samples (in triplicate) consisted of the same process without inoculation.

After fermentation, both control and fermented chia and sesame samples were heat-dried at 60 °C (Selecta J.P.200207, Germany) for 48 h until reaching a constant weight and milled (80 mm diameter grinding dial at 1980 rpm) for 3 min, resulting in control and fermented chia and sesame products with different structural properties. The control products had the appearance of grounded powders while the fermented sesame acquired the consistency of a paste (similar to tahini) and the fermented chia remained as a soggy powder (Figure S1).

### 2.3. In Vitro Simulated Digestion

The fermented and control chia and sesame products were subjected to a static in vitro digestion process, based on the standard protocol established by Brodkorb et al. (2019). The salivary fluid was obtained in vivo from a healthy volunteer, with the alpha-amylase being assessed as per Brodkorb et al. (2019) protocol [11]. The simulated gastric and intestinal fluids were prepared following the indications by Brodkorb et al. (2019) [11]. The digestions were simulated in 50 mL falcon tubes (in triplicate), which were highly agitated in the interior of a thermostated chamber at 37 °C (JP Selecta SA, Barcelona, Spain). For the oral stage, 5 g of the sample were mixed with the salivary fluid and vortexed for 1 min. Then, 10 mL of the simulated gastric fluid (2000 pepsin U/mL) was incorporated and the pH was adjusted with HCl 1M to 3, allowing for 120 min of the gastric stage. Finally, the intestinal stage was simulated for 120 min by adding 20 mL of the simulated intestinal fluid to the resulting chyme, which included a mixture of pancreatin (100 trypsin U/mL) and bile salts solution (10 mM in the final volume, bovine bile), and the pH was adjusted to 7 with NaOH 1 M. At the end of this stage, the tubes were immediately placed in ice to stop enzymatic reactions.

The described process was carried out three times: the first set of digestions was conducted to obtain the required aliquots for the lipolysis determination, for which the entire volume of digesta at the end of the intestinal stage was required. The second batch of digestion was used to obtain the aliquots for determining proteolysis (at intestinal times of 10, 30 and 120 min, 0.1 mL aliquots), polyphenols and antioxidant activity (0.3 mL aliquots at the end of the intestinal stage) and to calculate the matrix degradation index. The third set of digestions was carried out to measure particle size distribution and viscosity at the end of the intestinal stage.

### 2.4. Analytical Determinations

#### 2.4.1. Nutrient Profile

Lipid, protein, water and ash contents were determined by means of AOAC methodologies in control and fermented chia and sesame samples [12]. Carbohydrate content was estimated considering total solids and subtracting lipids, proteins and ashes.

#### 2.4.2. Fatty Acids Profile

Fatty acids in control and fermented chia and sesame samples were identified by gas chromatography mass spectrometry (GC-MS). The samples were first subjected to a Soxhlet extraction (AOAC, 2000) and then subjected to transesterification from fatty acids to methyl esters (FAMEs) with BF_3_ and methanol at 20 °C according to the IUPAC method. Then, samples were analyzed with an Agilent 5977A system and an HP-5MS UI (Agilent) (Column: 30 m × 0.25 mm, 0.25 μm film thickness) with helium as the carrier agent (1 mL/min). The method was followed according to Asensio-Grau et al. (2019) [13].

#### 2.4.3. Lipolysis Extent

The lipidic fraction of undigested and digested samples were analyzed by proton nuclear magnetic resonance (H^1^ NMR). Lipid extraction, spectra acquisition and quantification of lipolytic products were conducted according to Nieva-Echevarría et al. (2015) [14]. The number of moles of each molecule was calculated considering acyl groups by the equations previously validated by Nieva-Echevarría (2014) [15]. The NMR technique allows for quantifying triglycerides, partial triglycerides (monoglycerides and diglycerides) and free fatty acids.

#### 2.4.4. Proteolysis Extent

Proteolysis extent (%) was determined by the soluble protein fraction in the trichloroacetic acid (TCA) method, according to Asensio-Grau et al. (2020) [5]. TCA was mixed in a 12% (*w*/*w*) final concentration with the liquid fraction of digested samples. The mixture, after vortexing, was incubated for 15 min and centrifugated for 15 min at 8000 rpm. Then, the supernatant containing the soluble fraction in TCA (e.g., small peptides and aminoacids) was diluted with 50 mM EDTA and 8 M urea at pH 10, and protein content was determined by measuring absorbance at 280 nm against a prepared blank containing the digestion fluids. Proteolysis extent was expressed as grams of soluble TCA protein per gram of protein in the sample.

#### 2.4.5. Total Polyphenols and Antioxidant Activity

Total polyphenols were quantified according to the Folin-Ciocalteu method and antioxidant activity was determined by DPPH before and after digestion in fermented and non-fermented samples [5]. The hydrosoluble compounds were extracted by mixing the samples with methanol (80%) in non-digested (1:20 (*w*/*v*)) and digested samples; the proportion was 1:10 (*v*/*v*) in the bioaccessible fraction and 1:10 (*w*/*v*) in the solid fraction. The mixture was agitated at 55 rpm and 25 °C (Intelli-Mixer RM-2) for 2 h in darkness. Then, samples were centrifugated (Eppendorf Minispin) at 14 g-force and 20 °C for 20 min and supernatant was used to quantify polyphenol compounds and antioxidant activity.

For the determination of polyphenols, the methanolic extract (125 μL) was added to a 4 mL plastic cuvette with distilled water (0.5 mL) and the Folin-Ciocalteu reagent (125 μL). After 5 min, 1.25 mL of Na_2_CO_3_ (7% (*w*/*v*)) and distilled water (1 mL) were added, and absorbance was measured using a spectrophotometer (Helios Zeta UV/V is, ThermoScientific, Waltham, MA, UAS) at 760 nm. The calibration curve was made using gallic acid (0–500 mg gallic acid (GA)/L) as the standard, and polyphenols were expressed as mg of GA equivalents per gram of sample. To evaluate the effect of fermentation on the digestion of polyphenols, two different indexes were established: the recovery index and bioaccessibility. The recovery index (%) indicates the number of phenolic compounds available after intestinal digestion in the whole digesta (solid and bioaccessible fractions) compared to the initial concentration in the food before digestion (Equation (1)).

(1) $$\mathrm{Recovery}\;\mathrm{index}\;\left(\%\right)=\frac{\mathrm{polyphenols}\;\mathrm{in}\;\mathrm{the}\;\mathrm{whole}\;\mathrm{digesta}\;\left(\mathrm{mg}\right)}{\mathrm{polyphenols}\;\mathrm{in}\;\mathrm{the}\;\mathrm{sample}\;\left(\mathrm{mg}\right)}\times 100$$

Bioaccessibility (%) of polyphenols is defined as the percentage of phenols that are solubilized in the chyme after digestion (bioaccessible fraction) compared to the initial concentration in the food before digestion (Equation (2)).

(2) $$\text{Bioaccessibility}\;\left(\%\right)=\frac{\mathrm{polyphenols}\;\mathrm{in}\;\mathrm{the}\;\mathrm{bioaccessible}\;\mathrm{fraction}\;\left(\mathrm{mg}\right)}{\mathrm{polyphenols}\;\mathrm{in}\;\mathrm{the}\;\mathrm{sample}\;\left(\mathrm{mg}\right)}\times 100$$

#### 2.4.6. Particle Size

Particle sizes in control and fermented chia and sesame products, both before and after in vitro digestion, were measured by laser light scattering (Mastersizer 2000, Malvern, UK) by pouring ~5 mL of the sample in the wet route device of the equipment. The results were expressed as particle size distribution (in the range from 1 to 1000 µm) and as mean particle size diameter (d_4,3_).

#### 2.4.7. Matrix Degradation Index

The matrix degradation index (MDI) represents the proportion of solids that are released from the food matrix and mixed with the liquid fraction of the digesta. To calculate it, the total content of a digestion tube was centrifugated (4000 g-force for 20 min) and filtered with a metallic sieve (1.6 mm × 1.6 mm mesh) to separate out large particles (solid fraction) from the liquid part (bioaccessible fraction), which were placed in aluminum dishes and dried at 60 °C for 48 h and finally weighted. The MDI was expressed as the relation between the mass of the large particles and the mass of the sample prior to in vitro digestion [13].

#### 2.4.8. Viscosity

The apparent viscosity of the digesta belonging to control and fermented chia and sesame seeds was determined following the procedure reported by Lazaro et al. (2018) [16]. The determinations were conducted in triplicate with a rheometer (TA Instruments, Leatherhead, UK) using ~3 mL of the homogenized digesta (solid and liquid fractions) and a 40 mm 2° cone-plate geometry and a truncation gap of 50 µm, at 37 °C at a shear rate ranging from 0 to 300 s^−1^. The TA Instruments software was used to run the experiment and analyze the flow curves, which were fitted in the Power Law Model (Equation (3)), and to estimate the K (consistency index) and *n* (flow behaviour index) values.

σ = K × ˙γ*^n^* (3)

### 2.5. Statistical Analysis

Data were summarized as mean and standard deviation. Simple ANOVA analyses were performed to assess the statistical significance of fermentation and type of seeds on the undigested samples (nutritional composition, lipidic species, fatty acids profile and particle size), the effect of fermentation and type of seeds on digesta parameters (particle size, matrix degradation index and viscosity) and the effect of fermentation and type of seed on lipolysis, proteolysis and polyphenols bioaccessibility and antioxidant activity of digesta. Statgraphics Centurion was used, and the analyses were conducted with at least a significance of 95% (*p*-value < 0.05).

## 3. Results and Discussion

### 3.1. Effect of Solid-State Fermentation on Particle Size Distribution

**Figure 1:**
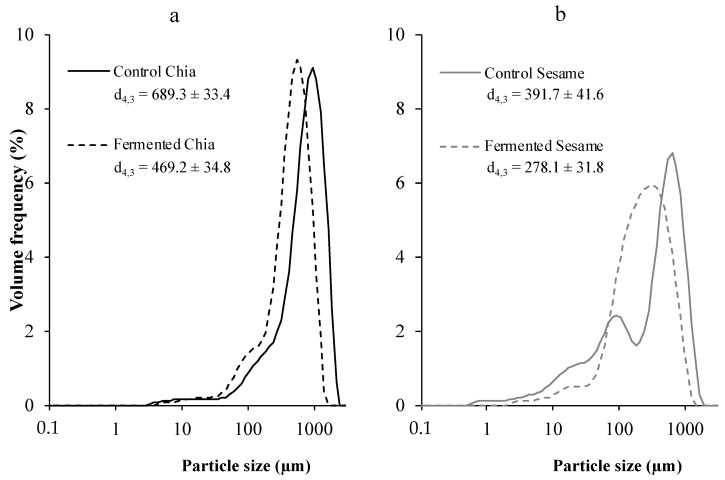
Particle size distribution and mean particle size (d_4,3_) in the non-digested control and fermented chia (**a**) and sesame products (**b**).

Fermentation led to significant differences regarding particle size in the products made out of chia and sesame and their fermented homonyms (Figure 1). Control chia had most of the volume frequency in particles close to 1000 µm, while fermentation resulted in a displacement of the distribution curve to smaller sizes. In the case of sesame, the control sample had a more heterogeneous pattern of particle sizes, ranging from 1 to 1000 µm, and fermentation was able to homogenize the distribution, depicting a spectrum with a peak between 100 and 1000 µm. Overall, fermentation induced a reduction in the particle size of the products, as evidenced by the d_4,3_ parameter. The achieved reduction could be attributed to the degradation or partial consumption of the substrates (chia and sesame) by the fungus. This detected structural change is highly relevant for explaining other results of the study because, as introduced above, the physical properties of the seeds are a major liming factor for the subsequent nutrient release and bioaccessibility. It has been repeatedly reported that reduction in the particle size of food substrates promotes nutrient digestibility [6,7,17]. In fact, previous studies, in which plant-based substrates were also fermented, dried and milled to produce flours, reported similar reductions in particle size compared to the non-fermented controls [5]. For the fermenting microorganism growth, some of the substrates in the plant cell walls of the grain are consumed, especially fibers and other carbohydrates [4]. The degradation of the external layer of the seeds as a consequence of fermentation could thus explain a facilitated structural breakdown in the milling process [4].

### 3.2. Effect of Solid-State Fermentation on Nutrient Profile

The fermentation process also accounted for modifications in the nutrient composition in the studied substrates (Table 1). Addressing first the comparison between chia and sesame products without fermentation, their nutrient profile differed, as sesame had less moisture, more lipids and slightly less protein. When comparing the composition before and after fermentation in both chia and sesame, similar changes were observed in terms of increased lipid and protein content, possibly at the expense of reduced moisture and carbohydrates [18]. In this sense, the most relevant changes in the fermented samples, compared to the controls, was the increase in lipid in both seeds (6.16% in chia and 15.38% in sesame), although protein also increased in chia.

The increase in protein content is in accordance with several previous studies applying solid-state fermentation on plant-based substrates. The rationale of this finding relies on the bioconversion of carbohydrates transforming the plant cell walls into fungal protein, which is a main component of the biomass of the growing *Pleurotus ostreatus* in the system [5]. However, few references in the literature report increased lipid content after fermentation. For instance, Mehdizadeh et al. (2015) reported increased total fat content in fermented rambutan seeds [19]. On the other hand, the fungus biomass, which accounts for approximately 5% of lipid fraction, could have also contributed, in part, to the increased total lipid content in the samples [20]. In addition, in the presence of a fat-rich substrate as chia and sesame, another possible explanation for increased total lipid content could be related to the de novo synthesis and accumulation of lipidic species. This is a documented phenomenon occurring when oleaginous microorganisms are able to utilize lipids in hydrophobic and lipid-rich substrates [21].

**Figure 2:**
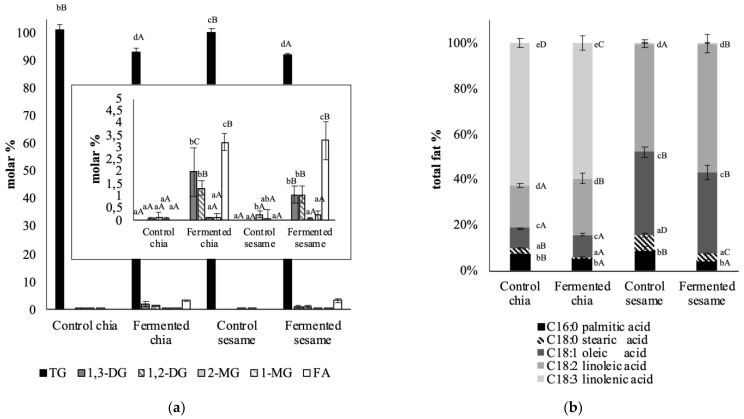
Characterization of lipids in chia and sesame products before (control) and after solid-state fermentation: (**a**) lipidic species and (**b**) fatty acid profile. ^a–e^ Letters refer to the homogenous groups obtained for different lipidic species (TG, 1,3-DG, 1,2-DG, 2-MG, 1-MG and FA) and type of fatty acid (C16:0, C18:0, C18:1, C18:2 and C18:3) at statistical significance of 95% (*p* < 0.05). ^A–D^ Letters refer to the homogenous groups obtained for different samples (control and fermented chia; control and fermented sesame) at statistical significance of 95 % (*p* < 0.05).

When looking into the lipidic species (Figure 2a), as expected, the control chia and sesame flours were composed solely of triglycerides with residual proportions of the other species, but fermentation accounted for the conversion of approximately 5% of the triglycerides into 1,3 and 1,2-diglycerides and fatty acids. This finding suggests that the fungus metabolism was able to use some of the lipidic fraction of the products as substrates. Indeed, Piscitelli et al. (2017) specifically assessed lipases produced by this fungus [22]. The study reported the optimal enzymatic activity conditions and the preferred substrates, concluding that at pH 6–8 and temperatures between 30 and 60 °C, *Pleurotus ostreatus* is able to hydrolyze triglycerides. Later, Rehman et al. (2019) addressed the potential of *Pleurotus ostreatus* for lipase production in SSF with different substrates, showing the highest activity of lipase was on canola oil seed cake, and concluding that when fermentation conditions are optimal, lipase activity can increase up to 1.6-fold [23]. Also, Chai et al. (2019) reported a high conversion rate of triglycerides into fatty acids after fermentation of rambutan seeds (4.3 folds) [24]. Considering the results of lipidic species in the present study’s samples, the incubation conditions during fermentation and/or the substrates used do not seem to be optimal for promoting the lipolytic activity of the lipases of *Pleurotus ostreatus*, as only 5% of triglycerides were hydrolyzed, compared to the 1–4-fold increases reported in other studies.

As shown, the lipid fraction of both chia and sesame represents the majoritarian component; therefore, characterization of the fatty acid profile (saturated or unsaturated), in addition to lipidic species, is a worthwhile task. Despite the fact that previous studies have established the composition of fatty acids in these seeds, scarce literature is available regarding the potential effect of fermentation on changing this profile in chia and sesame.

Focusing on control samples, chia and sesame products presented with some differences regarding the fatty acid profile (Figure 2b). Both had a low proportion of saturated fatty acids, but chia was richer in polyunsaturated fatty acids, especially linolenic (63.2 ± 2.2%), with linoleic being higher in sesame (47.1± 2.4%). These findings are in accordance with previous studies specifically assessing the free fatty acid profiles in chia [25] and in sesame [26]. The fermented counterparts resulted in some modifications in the relative concentration of fatty acids (Figure 2b), mainly related to a slight decrease in saturated fatty acids in favor of an increase of the unsaturated. Particularly, in chia, the content of saturated fat was reduced in approximately 3% from total fat content, and linolenic acid decreased in favor of increased linoleic (+6.5%) and oleic acids (+1%). In the case of sesame, only linoleic acid increased (+12%), showing a higher decrease in saturated fat (−7%) and oleic acid remaining unaltered.

These results are in accordance with previous research. One of the first studies assessing the change in free fatty acid profiles in oleaginous seeds as a consequence of fermentation focused on the African Oil Bean Seed, which presents with a similar total lipid fraction as chia and sesame [27]. In that study, after bacterial fermentation, palmitic acid was reduced from 5 to 4.5% of the total lipid content, stearic from 4.3 to 3.4% and linoleic from 42.8 to 40.9, with linolenic and oleic being those increasing around 2% [27]. The study concluded that the slight change in the fatty acid profile was a result of the fermenting microorganisms’ metabolism. More recently, Chai et al. (2019) addressed the study of solid-state fermentation of rambutan fruit presenting with a fat content of around 40% [24]. The determination of the fatty acid profile after 10 days of natural fermentation (type of microorganisms involved not specifically reported), conducted with a similar methodology as in the present study, reported similar findings as those previously described: an increase in total unsaturated fatty acids (from 54.9 to 84.5) at the expense of a decrease in saturated fat (from 45 to 14.5%). However, focusing on specific fatty acids, and unlike the results presented by Achinewhu (1986), the largest increase occurred in linoleic acid (2.3 to 24.1%), with arachidic acid (C20:0) showing the largest decrease (from 34.7 to 4%). A plausible explanation for the change in the fatty acid profile relates to the preference of some saturated fatty acids for the fermenting microorganisms as energy sources. From a different point of view, Luma Khairy et al. (2017) attributed the altered fatty acid composition in the seeds after fermentation to the use of some specific fatty acids to the development of their plant cell wall phospholipid development [28]. In addition, Dos Santos-Oliveira et al. (2011) identified a similar change in the fatty acid profile after fermenting rice bran with Rhizopus oryzae, accounting for decreased saturated and 5% increased unsaturated fatty acids, and a concomitant increase of phospholipids and decreased triglycerides [29]. These authors related the excess of lipids in the substrate to their use by the microorganism to produce more phospholipids, which are generally rich in unsaturated fatty acids. Moreover, Tzirita, Quilty & Papanikolaou (2020) note that the oleaginous microorganisms, which are able to use lipids as substrates, may produce de novo lipidic species with a polyunsaturated profile [21]. Overall, the results of the present study relate to an increase especially in linoleic acid, which could likely be related to the fact that this fatty acid represents around 60–75% of the fat fraction of the fungus [20]. Therefore, part of the increase in this fatty acid in the fermented products could be attributed to the biomass of the fungus in the final product.

In spite of some specific differences in terms of fatty acid profile after fermenting different plant-based substrates, even with different microorganisms, a common trend is the increase of unsaturated fatty acids along with a decrease in those with a saturated carbon chain. Therefore, the application of fermentation to edible seeds is supported to enhance the lipidic profile. This is of special relevance when substrates are rich in fat, such as chia and sesame, as the final product would result in higher content of polyunsaturated fatty acids. This type of fat is restricted to few dietary sources and is not frequently consumed by the general population. While saturated fats are needed for biological functions (e.g., structure of the cell walls), their dietary intake should be restricted to <10% of total daily energy intake, as higher consumptions are associated with the risk of developing obesity, diabetes or coronary disease, among other pathologies. On the other hand, according to the World Health Organization, the daily consumption of polyunsaturated fatty acids should be promoted and preferred, as these types of structures are needed for relevant functions, such as intervention in anti-inflammatory responses, among others [30].

Finally, another change in the composition of seeds after fermentation deserving attention is related to antioxidant activity (Table 2). Despite previous research reporting increased polyphenol bioaccessibility and antioxidant activity in fermented plant substrates compared to their controls [5], the present study was conducted on seeds, which unlike other substrates such as lentils or other legumes or cereal, contain high amounts of lipids. The fermentation process could have increased the release of lipids from the interior of the plant cells, thereby increasing their exposure to the environmental conditions, including oxidation. This phenomenon is especially relevant when the majoritarian fatty acids in chia and sesame are polyunsaturated and thus more sensitive to oxidation. In addition, lipids are present in large proportion in the study substrates. Therefore, a plausible explanation for the non-increased polyphenols and antioxidant activity is that these were consumed to protect against lipid oxidation [31,32] despite the fact that fermentation could have produced an increase of these compounds.

### 3.3. Matrix Degradation and Viscosity of the Digesta

During digestion, both mechanical forces and the enzymatic activity are the main factors contributing to matrix degradation and particle size reduction. However, the extent to which particle size is reduced largely depends on the characteristics of the food matrix being digested.

To quantify matrix breakdown, the matrix degradation index (MDI) has been used in some in vitro digestion studies [5,6]. As expected, fermented chia and sesame achieved higher MDI than their control counterparts (Table 2). The control sesame product reached significantly higher MDI than control chia (41.3 vs. 4.5%). However, both fermented products allowed for increased MDI compared to the control in a similar proportion (to 25% in fermented chia and 59% in fermented sesame). The rationale behind these results relates to the structural foundations discussed above to explain the change in the particle size. In the case of chia, the presence of fiber (>50%) and protein and the absence of carbohydrates in the pericarp are characteristic parameters of the structure [33], which could explain the higher difficulty in the hydrolytic action and subsequent degradation. In sesame, the lignified fibers in the parenchyma act as a resisting layer, the hydrolyzation of which enables the further effective seed dispersal [34]. It is therefore hypothesized that under the simulated digestion conditions in this study, the components of the parenchyma in seeds were more susceptible to hydrolysis than those in chia. In addition, the formation of the viscous layer in chia when mixed with the digestive fluids could have exerted a protective effect against enzymatic activity and mechanical forces.

Undertaking the process of in vitro digestion not only caused matrix breakdown, but along with it, digestion produced a change in the particle size of chia and sesame products. Considering d_4,3_ (volume weighted mean diameter) as a parameter to define this change, both control chia and sesame products achieved only a minor and non-significant reduction of particle size, while it was statistically significant in the case of the fermented samples (Table 2).

The almost unaltered particle size of control chia and sesame at the end of the intestinal digestion could be attributed to the physical properties of the plant cell walls in these matrices. They protect against physical and chemical degrading impacts, including the impossibility of enzymes or other agents to exert a disruptive action on it, therefore preserving the structure unaltered [6]. Despite the fact that the chia products had undergone a milling process, the particles corresponding to the pericarp, a very tight fibrous structure natively covering the chia seed [35], would have skipped breakdown during digestion due to the inability of enzymes to hydrolyze it. Conversely, the reduced particle size in the fermented matrices is related to the degradation of the components of this structure (lignins, glycans, and other fibers) which are utilized by the fungus for its growth [36]. This degradation, which facilitates the disintegration into smaller structures by digestion, did not only have an effect in the fermented and non-digested samples (as shown in Figure 1), but continued facilitating the matrix breakdown in the gastrointestinal digestion process (Table 2), as previously reported in other studies [5].

Other parameters defining the physical properties of the digesta that could interfere with nutrient hydrolysis are those related to viscosity. Previous in vitro digestion studies have associated highly viscous digestion media with decreased lipolysis, as it hinders the accessibility of lipases to their substrate: the fat globules that are present in the digestion medium [37]. The reason is that the increase in the viscosity of the digesta limits the mixing of the food matrix with the digestion fluids, thus reducing the chance that the enzymes will reach their substrates [38]. Similarly, viscosity in the digestion medium has been related to a decline of other nutrient hydrolysis apart from lipids [39]. Some food components have the ability to increase the viscosity of digestion media, especially water-soluble polysaccharides in the cell walls that can be released during digestion [7]. Another mechanism of polysaccharides or soluble fibers to inhibit macronutrient digestion is their disposal at the interface between the nutrient globule and the digestion medium, preventing the accessibility of enzymes [39].

**Figure 3:**
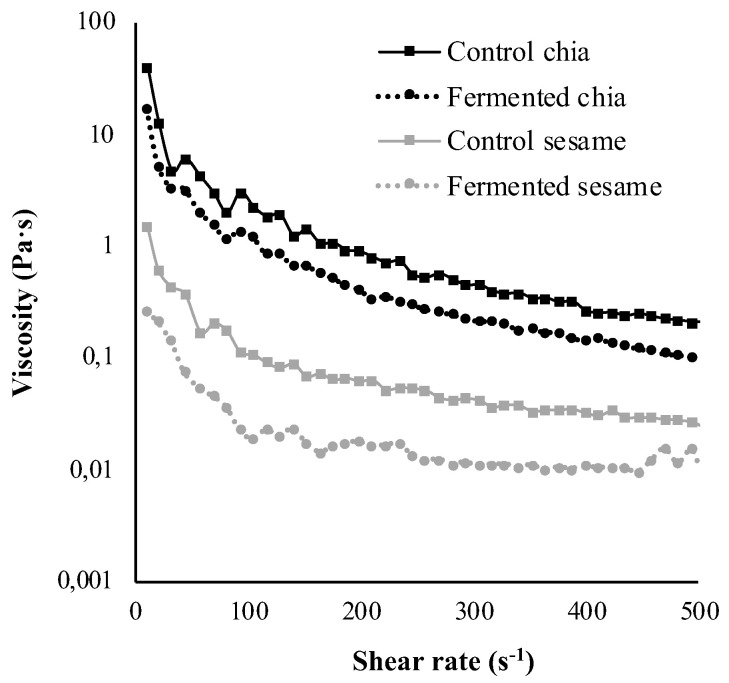
Viscosity of the digesta from control and fermented chia and sesame products as a function of the shear rate.

In this study, two high-in-fiber substrates were subjected to in vitro digestion; thus, assessing the viscosity they conferred to the digestion medium and the possible association with the extent of macronutrient digestibility was considered a relevant aspect to address. In chia, an important part relates to the mucilage, which represents 8.3% of the seed and is composed mainly of water-soluble polysaccharides [16]. It is well known that when chia seeds are soaked in water, a mucilaginous gel is exuded, which remains tightly bonded to the surface of the seed or nutlet [40]. The same phenomenon is observed when chia seeds or resulting particles from mincing chia seeds are submerged in digestion fluids [6]. Contrarily, sesame seeds lack a mucilage, so the gelation of the digestion fluids in which they are being digested does not occur. This difference between the two types of seed products in the viscosity conferred to the digestion media is observed in Figure 3, showing the flow curves of the intestinal digesta. Looking at the figure, all of the fluids showed shear-thinning behavior, as the apparent viscosity decreased with increasing shear rate. However, through the entire range, chia samples showed higher apparent viscosity than sesame samples (up to 102 difference), this result being attributed to the effect of the mucilage discussed above. In addition, the shear rate flow behavior results were fitted in the power law model (Table 2), following the procedure of Capitani et al. (2013) and Lázaro et al. (2018), who performed similar studies to this one [16,35]. In terms of consistence index (K) and flow behavior index (*n*) (i.e., the parameters of the power law), this difference between chia and sesame digesta were also made evident (Table 2). Another relevant observation was that the fermented samples depicted lower apparent viscosities than their corresponding control products. A possible explanation behind this could relate to reduced molecular weight of the polysaccharides dispersed in the digesta in the fermented samples (as reported in fermented cereals by Tsafrakidou et al., 2020) [41], which are known to be directly associated with the viscosity of the medium in which they are contained (Wood, 2002) [42].

When looking to a study assessing the viscosity of digesta samples in different isolated gums, similar rheological behaviors are comparable to the results of this study [43]. The assessed gums displayed flow curves of pseudoplastic fluids, and the curve belonging to locust bean gum was comparable to the one obtained for the control chia in this study. However, in that study, the parameters of the Power Law were very different than our results in chia, in terms of consistence index, which were in the range of 1.6 and 11.1 Pa.s. The higher values in this study could be attributed to the other particles present in the digesta, especially regarding the mucilage. On the other hand, the consistence index results for the sesame seeds are in the same range [43]. In another study focused on assessing the viscosity of digesta of chia mucilage in isolation, much lower Power Law parameters were obtained, despite the fact that the flow curves also indicated shear-thinning behavior. However, the amount of sample that was assessed was in the order of ten times lower than in the present study [16].

### 3.4. Macronutrient Digestibility

In order to assess lipid hydrolysis, the lipidic species in control and fermented chia and sesame products were determined before and after gastrointestinal digestion, in terms of triglycerides, diglycerides, monoglycerides and free fatty acids. Most lipids in foods are present in the form of triglycerides, which are molecules composed of a glycerol backbone to which three chains of fatty acids are attached by steric bonds [44]. Each anchoring point is known as the stereospecific position (sn), so triglycerides have three sn positions, with the sn-2 being the position in the middle. Pancreatic lipase is able to hydrolyze the triglyceride molecule by breaking the bond between the glycerol and the fatty acids. The literature reports more affinity for the sn-1 and sn-3 positions than for the sn-2, so the result of lipolysis is the release of two fatty acids and the resulting 1-monoglycerol, but the sn-2 position can also be hydrolyzed, thereby resulting in the release of three fatty acids [44].

Therefore, after 120 min of simulated intestinal digestion, a decrease in total triglycerides along with an increase in fatty acids and other intermediate species (mono and diglycerides) was to be expected. However, especially in plant-based foods, lipid release and digestibility rely on food matrix breakdown and the cell wall damage by the enzymatic and peristaltic forces, so that lipids can be released and digested [17,45]. This inherent resistance against digestion is also known as “the cell wall barrier” [7]. Indeed, for this reason, several plant-origin foods such as legumes or cereals undertake processing before ingestion, including different strategies that alter the physical structure and facilitate further bioaccessibility of nutrients. Even when the lipids are released, other components such as starch granules or protein can interact with lipids and prevent lipolysis [46]. On the other hand, the viscosity of the digestion medium has been mentioned before as a critical factor in the process of lipolysis.

**Figure 4:**
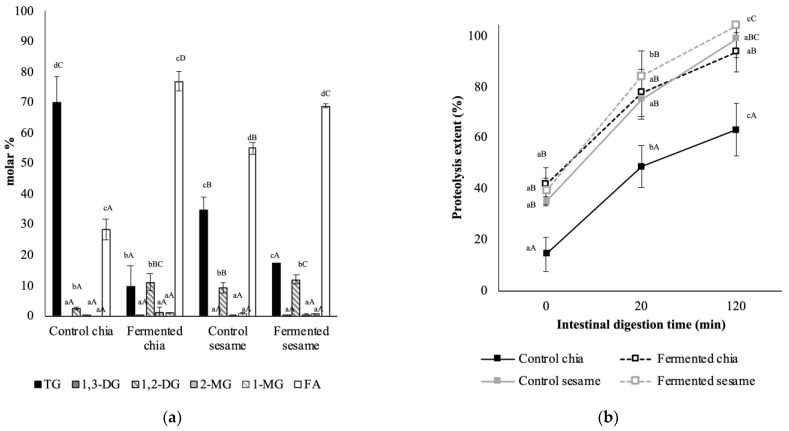
(**a**) Distribution of lipid species (molar percentage) in control and fermented chia and sesame products. Lipolysis extent is represented by the molar % of fatty acids and monoglycerols, these species being considered as the absorbable final products of lipolysis. ^a–d^ Letters refer to the homogenous groups obtained for different lipidic species (TG, 1,3-DG, 1,2-DG, 2-MG, 1-MG and FA) at statistical significance of 95% (*p* < 0.05). ^A–D^ Letter refer to the homogenous groups obtained for different samples (control and fermented chia; control and fermented sesame) at statistical significance of 95% (*p* < 0.05). (**b**) Progress and extent of proteolysis in control and fermented chia and sesame samples during intestinal in vitro digestion. ^a–c^ Letters refer to the homogenous groups obtained for different times (0, 20 and 120 min) at statistical significance of 95% (*p* < 0.05). ^A–C^ Letter refer to the homogenous groups obtained for different samples (control and fermented chia; control and fermented sesame) at statistical significance of 95% (*p* < 0.05).

In spite of the described factors preventing lipolysis in plant-based foods, fermentation allowed the products to show significantly higher digestibility than the control counterparts, which were in accordance with the limiting factors in plant-based foods (Figure 4a). Considering the extent of lipolysis as the molar percentage of fatty acids after digestion, and compared to the control samples, fermentation allowed for increasing lipolysis by 51% in chia seed flour and 16% in sesame seed products. The smaller proportion represented by monoglycerols could be also considered an absorbable fraction. This result can be supported from several perspectives. First, as shown in Table 2 above, fermented products produced less viscous digesta, thus the mixing of lipases with the released lipids from the matrices could be more effective than in the more viscous systems of the control products, leading to higher lipolysis. This could also explain the fact that sesame samples had better lipolysis results than chia samples. From another point of view, when looking at the particle size of the digesta in terms of d_4,3_, lipolysis was inversely proportional to this parameter, showing an R2 of 0.95. As previously argued, the surface area of the particles in the digestion medium determines the efficacy of the hydrolytic reactions. When the particle size is smaller, the surface area exposed to the digestion agents (pH, enzymes, bile salts, etc.) is increased, so the degradation of the matrix, including the breakdown of cell walls, is enhanced. This results in increased release of nutrients to the digestion medium, which eventually become more accessible to the enzymes [6].

There are scarce records in the literature addressing in vitro digestion of chia or sesame seeds or derivatives as a whole food matrix. The inalterability of seed structures against digestion, however, is a contrasted finding [6]. On the other hand, in the case of chia seeds, there are some studies assessing both: the effect of the chia fiber or mucilage in preventing digestion, and the study of lipolysis of isolated chia seed oil. The first group of studies agree that chia fiber or mucilage is successful in inhibiting macronutrient digestibility [47]. The other cited research line focuses on the lipid fraction of chia, which is rich in polyunsaturated fatty acids and thus has several potential benefits in health outcomes. Other studies assessing the behavior of digestion in isolated chia oil do not report results related to lipolysis [48]. Nonetheless, as shown in this study, the fermented, dried and minced chia seeds can achieve satisfactory lipolysis extents, so the negative impact of the matrix components on lipolysis would be minimized after solid-state fermentation with *Pleurotus ostreatus*.

The effect of fermentation on the digestion of protein was different in sesame compared to chia seeds’ flours. In Figure 4b, the progress of proteolysis at different intestinal digestion times is presented, along with the final extent which is considered as the result at 120 min. As expected, as in the case of lipolysis, fermentation of chia seeds led to products with enhanced proteolysis after in vitro digestion compared to controls (68 vs. 82%). This result is comparable to the improved proteolysis previously registered in fermented lentils with *Pleurotus ostreatus* to produce flours [5]. On the one hand, proteolysis could have increased in the fermented samples due to the breakdown of the plant cell walls, consumed as substrates for the fungus growth, which could have facilitated protein release to the digestion medium [5]. Moreover, the fermentation process accounts for bioconversion of the substrate components into fungal biomass, which increases as the fungus grows. Notably, the protein composition of *Pleurotus ostreatus* was reported to range between 13.1 and 16.9 g/100 g of biomass [49]. Therefore, in addition to the seeds’ protein, the fungal protein in the chia flour could be more easily hydrolyzed, as previously explained by Asensio-Grau et al. (2020) [5]. In a previous study assessing macronutrient digestibility in different chia structures, higher proteolysis was obtained for non-fermented chia flours (i.e., milled chia seeds) [6] than in the control chia product in the present study.

In contrast, proteolysis did not significantly change in the fermented sesame product compared to the control, as in both, it was close to 100% at the end of the intestinal stage. Limited information in the literature is available to explain this finding, as there are no studies specifically addressing the study of proteolysis in fermented or non-fermented sesame seeds. The explanation could be once more supported with the rheological characteristics of the digesta. In chia, the fermentation led to less viscous digesta, and proteolysis increased; in sesame, as viscosity was already low compared to chia in the digestion content of both fermented and non-fermented products, proteolysis was fully achieved. As previously discussed, the viscosity of the digestion medium can increase because of the presence of fibrous compounds, resulting in decreased enzymatic effective hydrolysis. In this sense, other authors have reported that fibers in the formulation of biscuits accounted for declined proteolysis during digestion [50].

### 3.5. Antioxidant Properties of the Digesta

The results in total polyphenols and antioxidant activity, both in the solid and in the bioaccessible fractions, are presented in Table 3. The total polyphenols in the bioaccessible fraction ranged between 11.54 and 17.09 mg of gallic acid equivalents per gram of sample, this result being in the order of ten times lower in the solid fraction. This proportion between the two fractions was similar to a previous study that focused on other polyphenol-rich substrates [51]. However, in the case of antioxidant activity, the difference between the solid and the bioaccessible fraction was not abrupt.

Focusing on the recovery index, fermentation led to increased values in chia after digestion. This fact could be attributed to the acidic and alkaline hydrolysis occurring at the stomach and small intestine, respectively [52]. Moreover, fermented samples and control sesame showed higher recovery index and bioaccessibility compared to the control chia. This finding could be related to the higher content of lipids in these matrices, as previous studies have related the influence of lipids on increased polyphenol acids during digestion [53]. These authors explained that lipids are emulsified by the bile salts resulting in lipid droplets, and anthocyanins, a type of phenol, can be incorporated into the lipid phase of these micelles. This fact could prevent their degradation during digestion, thereby increasing the polyphenol concentration in fatty food matrices.

Regarding the antioxidant activity in the bioaccessible fraction, results are comparable to the values registered in the samples prior to digestion, suggesting a preservation in the antioxidant activity throughout the digestion process. This finding is in accordance with previous studies that have reported maintained or even increased antioxidant activity after gastrointestinal digestion [54].

## 4. Conclusions

Fermentation of chia and sesame substrates has the potential to deliver products with increased lipid content, which in turn is richer in polyunsaturated fatty acids. The chemical composition and the physical changes accounted for by solid-state fermentation with *Pleurotus ostreatus* led to improved lipolysis and proteolysis, which were also related to the physical characteristics of the digesta. In conclusion, applying this biotechnological process on lipid-rich seeds seems to be a recommended approach to produce added-value products in terms of nutrient profile and digestibility. Further research is encouraged to assess the possible incorporation of this new product in the formulation of new foods.

## Figures and Tables

**Table 1:** Composition and antioxidant activity in chia and sesame products before (control) and after solid-state fermentation.

	Chia		Sesame	
	Control	Fermented	Control	Fermented
Moisture (g/100 g)	5.15 ± 0.10 ^b^	4.7 ± 0.2 ^a^	1.376 ± 0.005 ^b^	0.021 ± 0.002 ^b^
Protein (g/100 g)	22.5 ± 0.6 ^a^	25.69 ± 0.19 ^b^	21.00 ± 0.14 ^a^	21.8 ± 0.4 ^b^
Lipid (g/100 g)	29.95 ± 1.04 ^a^	36.11 ± 0.07 ^b^	42.42 ± 0.05 ^a^	57.8 ± 0.6 ^b^
Ash (g/100 g)	6.23 ± 0.91 ^a^	6.40 ± 0.23 ^a^	4.21 ± 0.06 ^a^	4.079 ± 0. 052 ^a^
Carbohydrate (g/100 g)	36.0 ± 0.4 ^b^	27.1 ± 0.3 ^a^	39.0 ± 0.2 ^b^	16.3 ± 0.4 ^a^
Polyphenols (mg GA eq/g sample)	30 ± 2 ^b^	15.78 ± 0.13 ^a^	11.7 ± 0.7 ^a^	14.03 ± 3.17 ^a^
Antioxidant activity (mg TX/g sample)	59.8 ± 0.3 ^b^	39.0 ± 0.2 ^a^	31.1 ± 0.9 ^b^	28 ± 2 ^b^

^a,b^ Letters refer to the homogenous groups obtained for different samples (control and fermented chia; control and fermented sesame) at statistical significance of 95% (*p* < 0.05).

**Table 2:** Physical characteristics of the digesta: matrix degradation index (MDI %), mean particle size (d_4,3_), and Power Law parameters: K (consistence index) and *n* (flow behaviour index).

	MDI (%)	d_4,3_	K (Pa·s)	*n*
Control chia	4.5 ± 0.06 ^a^	689 ± 15 ^d^	261.1	0.1821
Fermented chia	28.2 ± 3.7 ^b^	469 ± 12 ^c^	196.1	0.0901
Control sesame	42.18 ± 1.15 ^c^	391 ± 23 ^b^	12.01	0.0263
Fermented sesame	60.7 ± 2.4 ^d^	278 ± 34 ^a^	3.87	0.0052

^a–d^ Letters refer to the homogenous groups obtained for different samples (control and fermented chia; control and fermented sesame) at statistical significance of 95% (*p* < 0.05).

**Table 3:** Total polyphenols, polyphenols bioaccessibility and antioxidant activity in the non-digested and the bioaccessible of samples after in vitro digestion, both in control and fermented chia and sesame samples.

	Chia		Sesame	
	Control	Fermented	Control	Fermented
Total polyphenols in the bioaccessible fraction (mg GA eq./g sample)	17.09 ± 3.26 ^c^	13.54 ± 0.25 ^ab^	12.87 ± 1.38 ^a^	11.54 ± 0.28 ^a^
Total polyphenols in the non-digested fraction (mg GA eq./g sample)	2.60 ± 0.09 ^a^	3.33 ± 0.04 ^bc^	3.03 ± 0.36 ^b^	3.14 ± 0.17 ^b^
Polyphenols bioaccessibility (%)	55.8 ± 10.6 ^a^	85.8 ± 1.6 ^b^	110 ± 12 ^c^	82 ± 2 ^b^
Polyphenols recovery index (%)	64.06 ± 6.62 ^a^	85.78 ± 0.92 ^b^	136 ± 7 ^d^	108 ± 5 ^c^
Antioxidant activity in the bioaccessible fraction (mg TE/g sample)	40 ± 2 ^ab^	35 ± 8 ^a^	32.1± 2 ^a^	26 ± 7 ^a^
Antioxidant activity in the non-digested fraction (mg TE/g sample)	28 ± 3 ^c^	17 ± 2 ^ab^	19 ± 2 ^b^	15.5 ± 0.7 ^a^

^a–c^ Letters refer to the homogenous groups obtained for different samples (control and fermented chia; control and fermented sesame) at statistical significance of 95% (*p* < 0.05).

## Supplementary Materials

The following are available online at https://www.mdpi.com/article/10.3390/foods11030410/s1, Figure S1: Visual appearance of the obtained products: (**a**) control chia; (**b**) fermented chia; (**c**) control sesame; (**d**) fermented sesame.
