# Supplementary material for: Exploring the Impact of Solid-State Fermentation on Macronutrient Profile and Digestibility in Chia (Salvia hispanica) and Sesame (Sesamum Indicum) Seeds
Source: Foods. 2022 Jan 30;11(3):410. doi: 10.3390/foods11030410 (PMC8834584; doi:10.3390/foods11030410)
Supplement: Supplementary file 1 [file foods-11-00410-s001.zip › foods-1566259-supplementary.pdf]

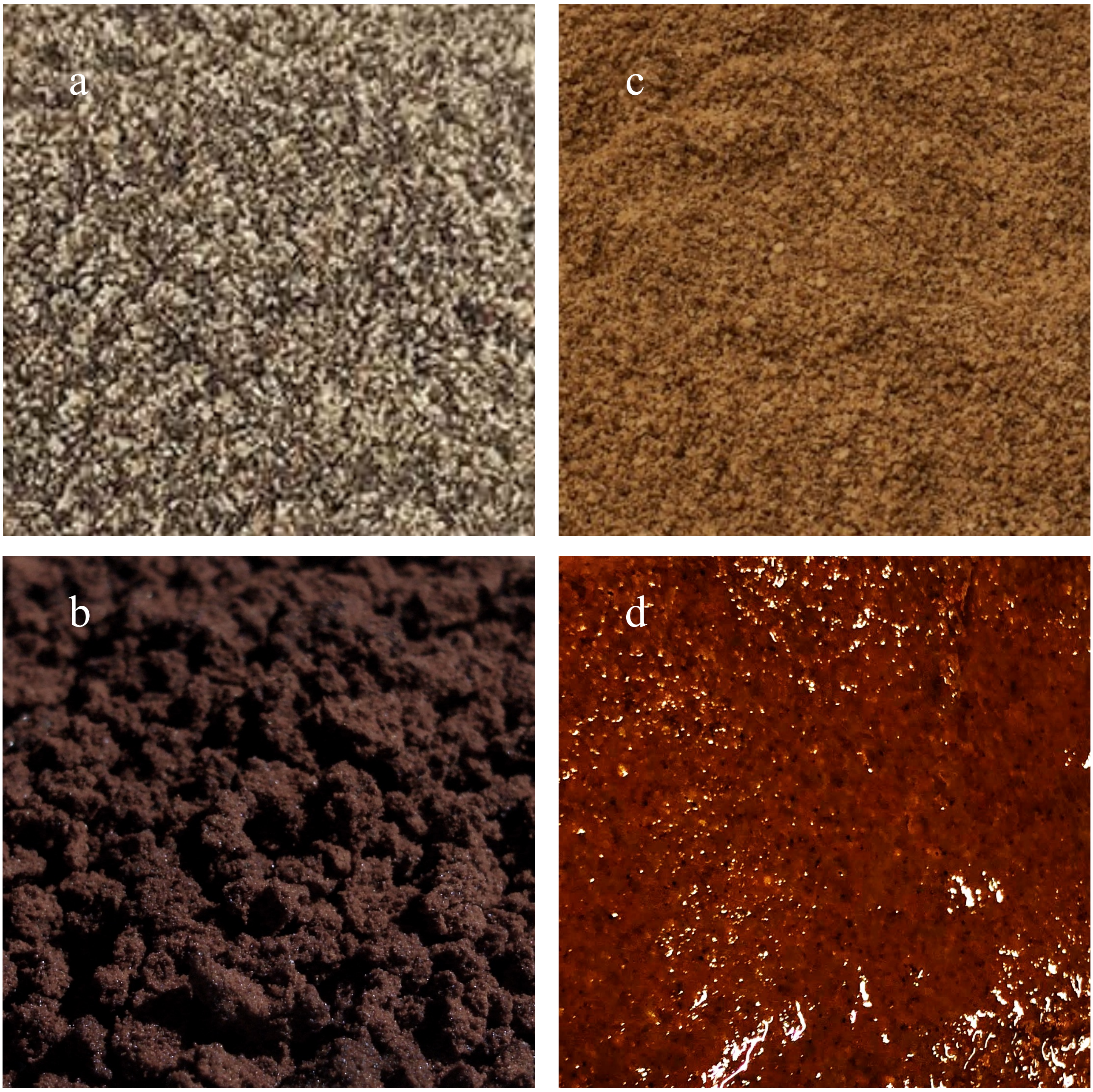

Figure S1. Visual appearance of the obtained products: (a) control chia; (b) fermented chia; (c) control sesame; (d) fermented sesame
